# Lipoic Acid Prevents High-Fat Diet-Induced Hepatic Steatosis in Goto Kakizaki Rats by Reducing Oxidative Stress Through Nrf2 Activation

**DOI:** 10.3390/ijms19092706

**Published:** 2018-09-11

**Authors:** Cristina Maria Sena, Maria Augusta Cipriano, Maria Filomena Botelho, Raquel Maria Seiça

**Affiliations:** 1 Institute of Physiology, Faculty of Medicine, University of Coimbra; Azinhaga de Santa Comba, Celas, 3000-548 Coimbra, Portugal; rmfseica@gmail.com; 2 iCBR, Faculty of Medicine, University of Coimbra; Azinhaga de Santa Comba, Celas, 3000-548 Coimbra, Portugal; mfbotelho@fmed.uc.pt; 3 Pathological Anatomy, University Hospitals of Coimbra, 3000-457 Coimbra, Portugal; augustacipriano@gmail.com

**Keywords:** diabetes, steatosis, oxidative stress, α-lipoic acid, TNF-α, Nrf2 levels

## Abstract

Prevention of hepatic fat accumulation may be an important approach for liver diseases due to the increased relevance of hepatic steatosis in this field. This study was conducted to investigate the effects of the antioxidant α-lipoic acid (α-LA) on hepatic steatosis, hepatocellular function, and oxidative stress in a model of type 2 diabetes fed with a high fat diet (HFD). Goto-Kakizaki rats were randomly divided into four groups. The first group received only a standard rat diet (control GK) including groups 2 (HFD), 3 (vehicle group), and 4 (α-LA group), which were given HFD, ad libitum during three months. Wistar rats are the non-diabetic control group. Carbohydrate and lipid metabolism, liver function, plasma and liver tissue malondialdehyde (MDA), liver GSH, tumor necrosis factor-α (TNF-α) and nuclear factor E2 (erythroid-derived 2)-related factor-2 (Nrf2) levels were assessed in the different groups. Liver function was assessed using quantitative hepatobiliary scintigraphy, serum aspartate, and alanine aminotransferases (AST, ALT), alkaline phosphatase, gamma-glutamyltranspeptidase, and bilirubin levels. Histopathologically steatosis and fibrosis were evaluated. Type 2 diabetic animals fed with HFD showed a marked hepatic steatosis and a diminished hepatic extraction fraction and both were fully prevented with α-LA. Plasma and liver tissue MDA and hepatic TNF-α levels were significantly higher in the HFD group when compared with the control group and significantly lower in the α-LA group. Systemic and hepatic cholesterol, triglycerides, and serum uric acid levels were higher in hyperlipidemic GK rats and fully prevented with α-LA. In addition, nuclear Nrf2 activity was significantly diminished in GK rats and significantly augmented after α-LA treatment. In conclusion, α-LA strikingly ameliorates steatosis in this animal model of diabetes fed with HFD by decrementing the inflammatory marker TNF-α and reducing oxidative stress. α-LA might be considered a useful therapeutic tool to prevent hepatic steatosis by incrementing antioxidant defense systems through Nrf2 and consequently decreasing oxidative stress and inflammation in type 2 diabetes.

## 1. Introduction

Liver steatosis (fatty liver) is frequently observed in conditions such as obesity and diabetes [1]. Although often considered as a benign condition, steatosis may evolve to steatohepatitis and even to cirrhosis. Lipid peroxidation has been implicated in non-alcoholic steatohepatitis (NASH). Based on studies in animal models, it was previously described that lipid peroxidation is one of the mechanisms that could promote the progression from steatosis to steatohepatitis, which is a process characterized by inflammation [1,2]. Lipid peroxidation can be demonstrated in steatosis but progression of steatosis to steatohepatitis in humans is not a constant feature, which implies the requirement of other factors in the pathogenesis of this process [3].

The pathogenesis of NASH remains elusive. It has been previously shown that a high-fat diet (HFD), insulin resistance, inflammation, and oxidative stress are considered important in the pathogenesis of human NASH [4,5].

There is not yet a drug therapy for NASH. TNF-α is a pivotal pathogenic factor playing a role in almost all steps of NASH pathogenesis along with oxidative stress [5]. Therapeutic options targeted against TNF-α appear as rational treatments of NASH. In addition, decrementing oxidative stress and strengthening the antioxidant defense systems is another viable option.

α-Lipoic acid is found to be naturally occurring as a prosthetic group in alpha-keto acid dehydrogenase complexes of the mitochondria playing a crucial role in metabolism. It has been used as therapy for many diseases associated with impaired energy utilization such as type 2 diabetes [6]. α-LA has also triglyceride-lowering properties that have been recognized in animal models [7,8] and in humans [9]. In addition, α-LA has been known to attenuate liver damage related to alcohol abuse, metal intoxication, and mushroom and CCl_4_ poisoning [10,11,12]. It can act as a potent antioxidant and ameliorate oxidative stress both in vitro and in vivo [13,14]. However, the effect of α-LA on HFD fed GK rats has not yet been investigated at the hepatic level. In the present study, we aimed to investigate the effect of decrementing oxidative stress with α-LA on HFD induced dyslipidemia and unravel the impact of oxidative stress on hepatic steatosis in diabetic GK rats. Serum biochemical parameters, systemic and hepatic oxidative stress, and liver inflammatory parameters, histology, the antioxidant defense system, and nuclear Nrf2 activity were measured in GK rats fed a normal diet and a HFD with or without α-LA and compared with normal nondiabetic Wistar rats.

## 2. Results

### 2.1. Animal Characteristics

Food consumption and water intake did not significantly change over the experimental period between the different groups studied [15]. Fifteen-month old GK rats were initially matched with regard to levels of glucose and subsequently divided in groups. Physical and metabolic parameters concerning the above animals are shown in Table 1. GK rats typically exhibit an inferior body weight compared to age-matched Wistar rats. Feeding the HFD for three months to GK rats significantly increased body weight compared to control GK rats. Livers from the GK rats fed with HFD were significantly greater in size compared to control GK rats and significantly smaller in the α-LA-treated GK rats compared to the GK fed with HFD. Adjusting liver weight to body weight, these characteristics did not change except for the GK group that presented a lower liver weight when compared to Wistar rats but, with the adjustment, this feature is lost (Table 1). Fasting occurred 2 h after load glucose levels were higher in GK rats when compared to age-matched Wistar values. Fasting glucose was significantly reduced after α-LA treatment (Table 1). Control GK rats had normal levels of cholesterol and triglycerides and HFD induced an increment in the levels of triglycerides as well as total and non-HDL cholesterol in GK rats (Table 1). Soybean oil (SO) and α-LA treatment normalized serum levels of total cholesterol, triglycerides, and non-HDL cholesterol in GK rats (Table 1).

Serum aspartate aminotransferase (AST), alkaline phosphatase (ALP), and γ-glutamyltranspeptidase (γGT) levels were significantly increased in the diabetic GK and HFD groups and alanine aminotransferase (ALT) levels were not significantly different. In addition, the ratio of AST/ALT was significantly increased in the group treated with soybean oil and reduced in α-LA treated GK rats due to the changes observed in the levels of ALT. Serum ALP levels were significantly reduced in GKHFD treated with SO and α-LA (Table 2) and γGT levels decreased only in the α-LA treated group (Table 2). Total bilirubin and albumin levels did not significantly change between the experimental groups (*p* > 0.05).

Hepatobiliary scintigraphy using iminodiacetic acid analogues labeled with ^99m^Tc has been applied in several animal models of liver disease to study hepatic function [16,17,18] through hepatic extraction fraction (HEF) calculation (Table 2). This parameter was significantly decreased in HFD fed GK rats and fully reverted in the GK groups treated with soybean oil and α-LA (Table 2).

### 2.2. Histology

**Figure 1:**
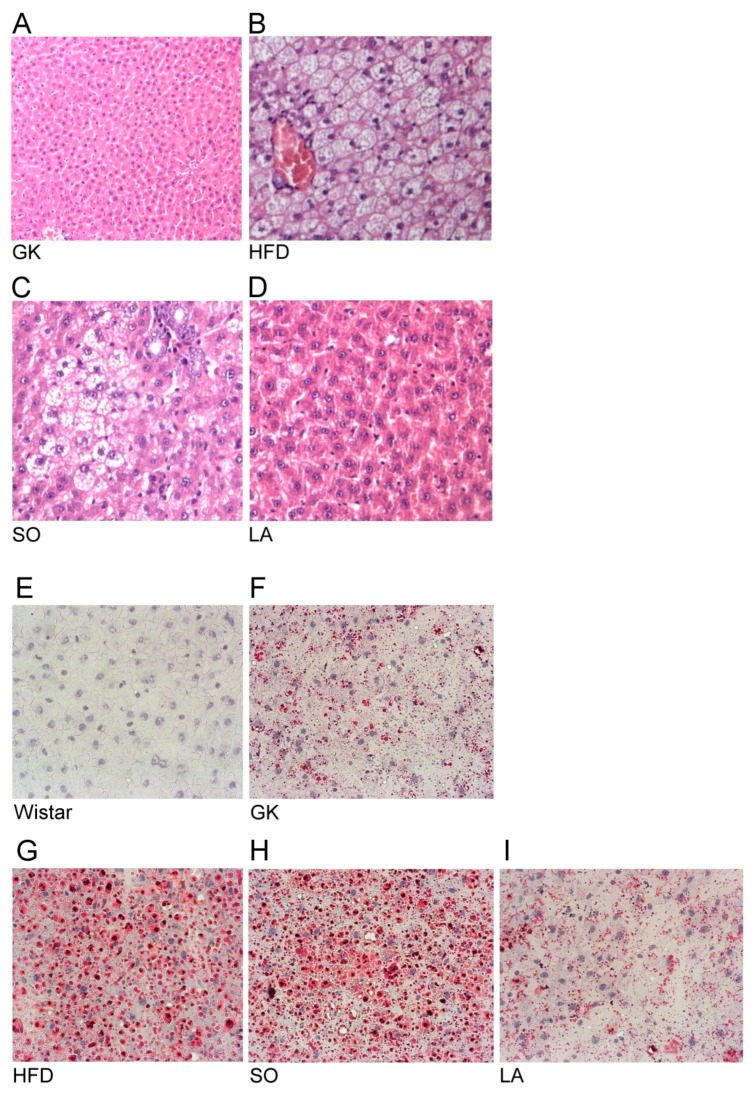
Effects of α-lipoic acid (α-LA) on liver steatosis. (**A**–**D**) H&E stained liver sections. Long-term histological outcome of Goto-kakizaki (GK) rats fed a high fat diet (HFD) with or without vehicle (soybean oil, SO) or α-LA (D) treatment compared with GK control (**A**, ×100). Normal liver architecture in the GK control group (**A**, ×100). After three months of HFD (**B**, ×200) a diffuse micro-vesicular steatosis was observed in the liver (hematoxylin-eosin, ×200) that persists in the vehicle treated group (SO, **C**, ×200). Note the prevention in steatosis in the α-LA treated group (**D**, ×200). (**E**–**I**) Oil Red O staining of Wistar, GK rats fed with normal or HFD with or without SO or α-LA treatment. Wistar group (**E**, ×200); GK control group (**F**, GK, ×200). After 3 months of HFD (**G**, ×200), an increased lipid accumulation was observed in the liver that persists in the vehicle treated group (**H**, SO, ×200). Note the decrement in lipid deposition (similar do the control GK group) in the α-LA treated group (**I**, LA, ×200).

As demonstrated in Figure 1A, hematoxylin and eosin staining showed normal liver architecture in the diabetic GK group (Figure 1A). The HFD and SO groups showed profound steatosis with microvesicular fat accumulation (Figure 1B,C respectively). Livers from rats fed HFD diets showed a micro-vesicular steatosis (Figure 1B) without fibrosis or necrosis [19] that persist after soybean oil (vehicle) treatment (Figure 1C). α-LA-treated GK rats displayed no signs of steatosis and this treatment was able to prevent HFD induced liver damage (Figure 1D).

To determine hepatic lipid deposition, oil-red O was also evaluated (Figure 1E–I). The HFD and SO groups showed marked steatosis with increased lipid accumulation (Figure 1G,H, respectively). α-LA-treated GK rats displayed a significant decrement in lipid deposition (similar do the control GK group). This treatment was able to prevent HFD induced liver damage (Figure 1I).

### 2.3. Intrahepatic Lipid Content

**Figure 2:**
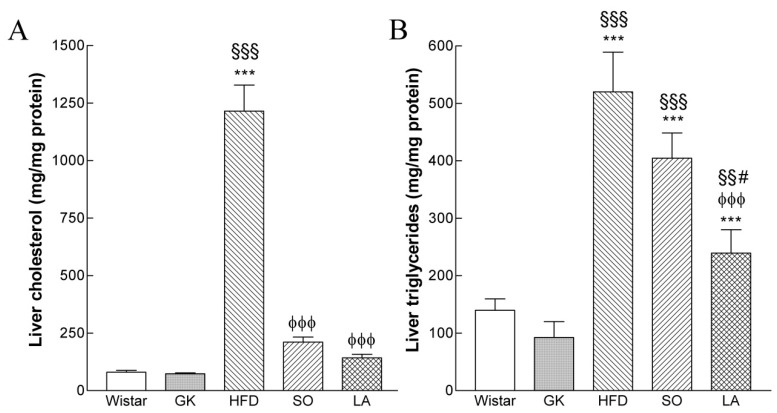
Effects of α-lipoic acid (α-LA) on hepatic cholesterol (**A**) and triglycerides (**B**) levels in Wistar, Goto-kakizaki (GK) rats control and GK fed a high fat diet (HFD) with or without vehicle (soybean oil, SO) or α-LA. Data are expressed as mean ± SEM (n = 7 animals per group). *** *p* < 0.001 vs. Wistar group, §§ *p* < 0.01, §§§ *p* < 0.001 vs. GK group, φφφ *p* < 0.001 vs. GKHFD group, # *p* < 0.05 vs. GK SO group.

At the end of treatment, liver cholesterol levels significantly increased in HFD group (15-fold) and decreased in SO and α-LA-treated GK groups (Figure 2A). Hepatic triglycerides were significantly increased in the HFD (4-fold) and HFD/SO group (3-fold) and decreased in α-LA-treated GK group (Figure 2B).

### 2.4. Effect of Lipoic Acid on Hepatic Antioxidant Enzymes

As shown in Table 3, the GK and HFD and SO groups showed a significant reduction in the hepatic activities of antioxidant enzymes when compared to age-matched Wistar rats. The GSH-dependent antioxidant enzymes activities GPx and GRd showed 21% and 33% reductions, respectively, in the HFD group when compared with the non-diabetic Wistar group. Antioxidant enzymes (GPx, GRd) in the α-LA-treated GK group were significantly increased when compared with those in the HFD group.

### 2.5. Oxidative Stress Biomarkers

Systemic oxidative stress biomarkers including plasma levels of MDA and urinary levels of 8-OHdG were significantly higher in diabetic GK rats and GKHFD (Table 3). Soybean oil treated rats also presented higher levels of oxidative stress parameters while α–LA treated GK rats displayed significantly lower levels of these parameters (Table 3). Serum uric acid levels were significantly higher in GK and GKHFD groups when compared to age-matched Wistar rats and α–LA treatment reduced this parameter to the levels observed in non-diabetic Wistar rats (Table 3).

### 2.6. Effect of Lipoic Acid on Liver Lipid Peroxidation

**Figure 3:**
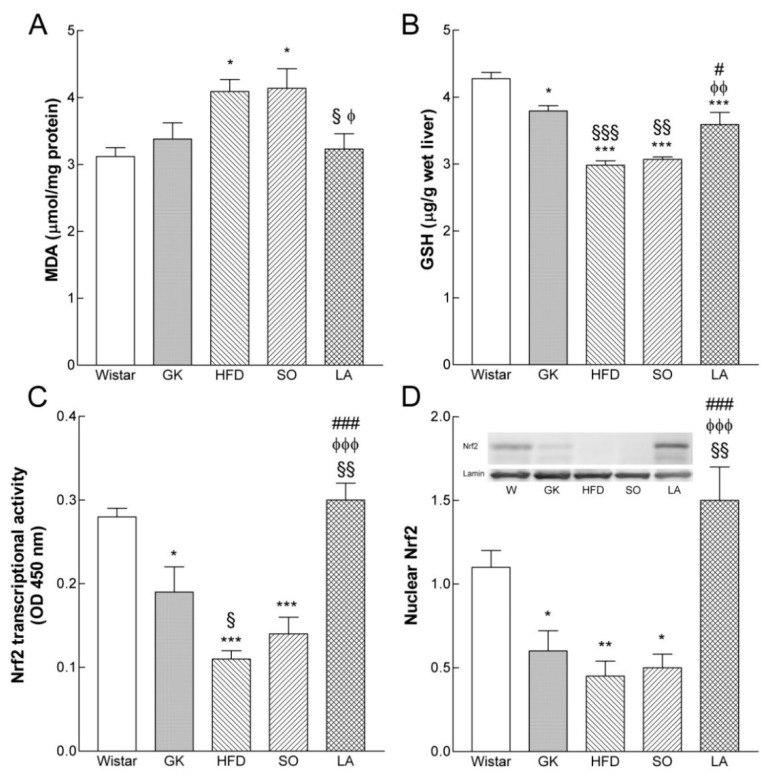
Effects of α-lipoic acid (α-LA) on lipid peroxidation (MDA), glutathione (GSH), and Nrf2 levels in the liver of Goto-kakizaki (GK) rats fed a high fat diet (HFD) compared with nondiabetic Wistar rats. Diabetes and HFD-induced impairment in the nuclear accumulation of Nrf2 and an increment in lipid peroxidation with concomitant reduction in GSH levels and α-LA prevented these effects. (**A**) Liver malondialdehyde (MDA) levels, (**B**) liver GSH levels, (**C**) the nuclear extracts depicted on panel D were also used for the measurement of Nrf2 DNA-binding activity using a TransAm Binding Assay (Active Motif), (**D**) representative Western blot analysis and average densitometry data (normalized with lamin values) of nuclear Nrf2 protein expression in livers of Wistar, Goto-kakizaki (GK) rats control and GK fed with high fat diet (HFD) with or without vehicle (soybean oil, SO) or α-LA. Data are expressed as mean ± SEM (n = 7 animals per group). * *p* < 0.05, ** *p* < 0.01, *** *p* < 0.001 vs. Wistar group, § *p* < 0.05, §§ *p* < 0.01, §§§ *p* < 0.001 vs. GK group; φ *p* < 0.05, φφ *p* < 0.01, φφφ *p* < 0.001 vs. GKHFD group, # *p* < 0.05, ### *p* < 0.001 vs. GK SO group.

Liver tissue MDA levels were significantly higher in the GK group fed with HFD and in the group treated with the vehicle (SO) when compared to the control Wistar group. Noteworthy, in the α-LA-treated GK group liver tissue, MDA levels were decreased when compared to the GK and HFD groups (Figure 3A).

### 2.7. Effect of Lipoic Acid on GSH Levels

The livers of diabetic GK rats presented a significantly lower GSH content, which was further reduced by HFD. The GSH content in the α-LA treated GK group was significantly increased when compared with the HFD and SO groups (Figure 3B).

### 2.8. Effect of Lipoic Acid on Hepatic Nrf2 Levels

We next examined whether hepatic Nrf2 levels are affected by diabetes and HFD. Western blot analyses in liver tissues revealed that total Nrf2 protein levels were not significantly different between the groups. Western blot evaluation showed that hepatic nuclear Nrf2 levels declined by 30% in GK rats fed with HFD (Figure 3D). In addition, we found that α-LA potently increases hepatic nuclear Nrf2 levels, which leads to the activation of downstream mechanisms that probably promote GSH synthesis.

We also examined Nrf2 signaling by measuring the rates at which Nrf2 binds to the ARE in liver tissue of control age-matched Wistar rats and the different groups of GK rats. Nrf2:ARE binding was 30% lower in diabetic GK rats when compared with age-matched Wistar rats (Figure 3C). Nrf2:ARE binding was significantly increased in the α-LA treated GK group when compared to the HFD group (Figure 3C).

### 2.9. Hepatic TNF-α Levels

**Figure 4:**
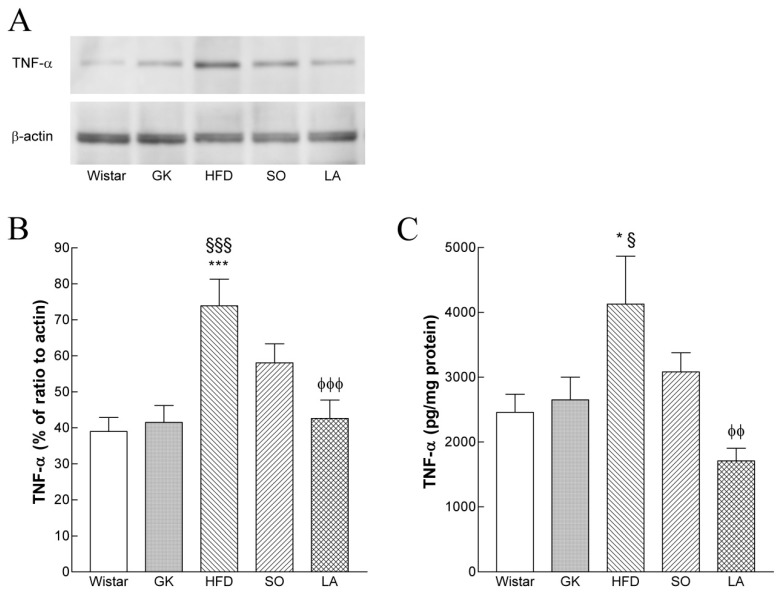
Effects of α-lipoic acid (α-LA) on tumor necrosis factor-α (TNF-α) levels in the liver of Goto-kakizaki (GK) rats fed a high fat diet (HFD) compared with nondiabetic Wistar rats. (**A**) representative Western blot analysis of TNF-α levels. (**B**) Average densitometry data (normalized with β-actin values) expressed as a percentage. (**C**) Protein TNF-α levels in livers of Wistar, Goto-kakizaki (GK) rats control and GK fed a high fat diet (HFD) with or without vehicle (soybean oil, SO) or α-LA. Data are expressed as mean ± SEM (n = 7 animals per group). * *p* < 0.05, *** *p* < 0.001 vs. Wistar group, § *p* < 0.05, §§§ *p* < 0.001 vs. GK group, φφ *p* < 0.01, φφφ *p* < 0.001 vs. GKHFD group.

ELISA and Western blot analysis revealed that hepatic TNF-α levels in the HFD group were significantly higher than in the Wistar and GK control groups. However, in the α-LA-treated GK group, hepatic TNF-α levels significantly decreased when compared to the HFD group (Figure 4A,B).

## 3. Discussion

Diabetic GK rats fed with HFD developed typical histopathologic micro-vesicular steatosis accompanied by an increment in hepatic triglycerides and cholesterol and a reduction in hepatic extraction fraction. Additionally, systemic and liver markers of oxidative stress and inflammation were increased in GKHFD rats and a decrement in liver Nrf2 activation was observed to be associated with reduced hepatic GSH content and decremented activities of the antioxidant enzymes (GPx and GRd). Treatment with α-LA significantly prevented the effects of HFD not only by reducing hepatic TNF-α but also suppressing the oxidative stress markers. α-LA decrements of oxidative stress through activation of Nrf2 leads to an increment in antioxidant defense mechanisms. Consequently, inflammation is reduced and, globally, these effects reduce hepatic steatosis. Our study strongly suggests that α-LA might serve as a therapeutic tool to attenuate hepatic steatosis.

Many studies indicated that obesity and a metabolic syndrome are associated with steatosis [1,3]. Accordingly, our animal model developed marked steatosis only after feeding HFD. GK rats exhibit a spontaneous polygenic form of diabetes and are a commonly used animal model for diabetes studies. Elevated blood glucose and peripheral insulin resistance are common features of these animals [15,20]. They exhibit mild hyperglycemia at fasten and increased gluconeogenesis [20]. They have a non-obese phenotype ideal for the study of diabetes without obesity. Chronic high fat feeding will exacerbate diabetes and its complications, promote dyslipidemia, and cause buildup of triglycerides in the liver leading to steatosis.

Concerning the vital role of oxidative stress in the etiology of liver injury, we studied the efficacy of α-LA, which is a naturally occurring compound with a powerful in vivo antioxidant activity, in the prevention and treatment of liver disease in an animal model of type 2 diabetes. α-LA can modulate the redox status of cells and the activities of proteins. This process affects cell signaling and transcriptional responses involved in glucose and lipid metabolism [21]. It was previously described that α-LA improved liver transaminases [22], enhanced scavenging of reactive oxygen species, increased activities of antioxidant enzymes leading to a reduction in oxidative stress and inflammatory signals, decrement in DNA damage, suppressed the fibrotic process, and improved lipid metabolism [10,21]. Importantly, in this study, LA exerts its effects through a mechanism that does not interfere with body weight. It is frequently used to observe in other reported studies a decrement in obesity and, therefore, a concomitant improvement in overall liver metabolism.

In the present study, α-LA was given as a supplement to aged GK rats fed a high fat diet. The range of doses used in the present study was consistent with those in other studies on the effect of α-LA on rats [15,23]. We found that feeding α-LA for three months suppressed the increment of liver weight induced by HFD (Table 1), normalized the lipid profile and hepatic extraction fraction, and improved liver transaminases.

Accordingly, a chronic α-LA supplement prevented nonalcoholic fatty liver disease in Otsuka Long-Evans Tokushima Fatty rats through multiple mechanisms by reducing steatosis, oxidative stress, immune activation, and liver inflammation [24].

In addition, soybean oil, which is the vehicle of α-LA in this study, had a significant effect in reducing systemic cholesterol and triglycerides levels without effects on oxidative stress, inflammation, and micro-vesicular steatosis. In agreement, we [15] and others reported similar effects. It has been reported that constituents of soybean oil such as α-linolenic acid are able to decrement cholesterol levels in humans [25]. Our results show a decline in transcriptional activity of Nrf2 in the liver of the GK group fed with HFD that leads to a decrement in glutathione levels, which is reversible with lipoic acid treatment. It was recently described that α-LA exerts its antioxidant effect via Nrf2 activation in methotrexate-treated rats [26]. In addition, it was previously shown that α-LA was able to induce the de novo synthesis and regeneration of GSH by activating the transcriptional factor Nrf2 [12,27]. In hepatocytes, Nrf2 is also an essential early player in the rescue of oxidative stress by α-LA, which leads to protection against lipoapoptosis [7]. It was previously observed that nuclear Nrf2 levels declined in the liver of aged rats. However, old rats receiving R-LA (40 mg/kg b.w. i.p.) displayed significant increases in glutamine-cysteine ligase activity and GSH concentration in the liver up to the levels found in young control animals through a mechanism that involves Nrf2. Nrf2 expression and activation is reduced in the liver with histological criteria of NASH [28]. In addition, previous studies indicated that Nrf2 is functionally involved in lipid deposition in the liver. It was described that, basally, Nrf2 regulates a number of proteins involved in the synthesis and metabolism of fatty acids and other lipids [29]. Using potent activators of Nrf2, triterpenoids (lipid soluble molecules synthesized from oleanolic acid), Shin and co-workers have shown a reduced accumulation of lipids in the livers of mice on a high fat diet via the Keap1/Nrf2 pathway [30].

GSH significantly declines in the liver of aged and type 2 diabetic animal models. A decrease in the level of glutathione in type 2 diabetes [31] is in agreement with the present study. The reduction in liver GSH content in diabetic GK rats may be caused by the increment in the oxidative stress. α-LA improves GSH levels in agreement with other studies [32]. Reduced GSH is important in normal cell membrane function as well as in other structural proteins. α-LA protects against oxidation incrementing cellular GSH content.

Nrf2 activation may also lead to a reduction in inflammation being protective against NAFLD and NASH [33]. In agreement, our work has shown a decrement in TNF-α levels in the group treated with α-LA.

In our study, it is particularly interesting that the normalizing effect of α-LA on liver cholesterol cannot exclude the involvement of other mechanisms independent of Nrf2. It was recently shown that Nrf1 was able to regulate both proteostasis and metabolism [34] as well as cholesterol homeostasis and NASH [35]. It is, therefore, a possible mechanistic approach that is worth exploring in future studies.

In addition, we show that uric acid levels are significantly increased in GK rats and GK fed with HFD. It is known that uric acid is an antioxidant extracellularly. Due to its intracellular effects, uric acid promotes oxidative stress in different cell types including hepatocytes [36]. In addition, uric acid also leads to fat accumulation in hepatocytes due to the inhibition of the rate-limiting enzyme in fatty acid oxidation [37]. Thus, uric acid also contributes to micro-vesicular steatosis observed in our animal model. Importantly, α-LA was able to reduce uric acid levels to the levels observed in Wistar rats.

Similar to α-LA, Alpinia zerumbet has antioxidant, anti-inflammatory, and hypolipidemic properties and it has been popularly recognized as an excellent hepatoprotector [38]. This medicinal plant has striking anti-aging and anti-obesity effects incrementing the lifespan in rodents and humans and deserves future research [39].

Our study strongly suggests that α-LA might serve as a therapeutic tool to attenuate hepatic steatosis by incrementing antioxidant defense systems through Nrf2 and consequently decreasing oxidative stress and inflammation in type 2 diabetes associated with hyperlipidemia. These findings suggest new strategies to prevent oxidative stress and hepatic steatosis with reduced hepatic function in diabetes associated with obesity. In order to conquer the growing epidemic of fatty liver disease, supplementation with α-LA is probably an important strategy to fight against liver disease.

## 4. Materials and Methods

### 4.1. Animals

Fifteen-month-old male Wistar and diabetic GK rats originated from our local colony in Coimbra (Portugal). Control rats were fed, *ad libitum*, with standard pellet chow (Diet AO4 Panlab) and allowed free access to water. Animals were kept in rooms with 12-h periods of light and darkness. The GK rats were divided in four groups: (1) control GK group fed a standard diet. (2) GK fed a high fat diet (HFD), 7.5% cocoa butter, and 1.25% cholesterol for 3 months. (3) GK fed with HFD and treated, 3 days/week for 3 months, with an intraperitoneal injection of soybean oil (2 mL Kg^−1^ body weight, the vehicle of α-LA). (4) GK fed with HFD and treated, 3 days/week for 3 months with an intraperitoneal injection of α-LA ((±)-α-lipoic acid, 50 mg Kg^−1^ body weight) in soybean oil [15]. At the end of treatment, urine and blood were collected. Animals were sacrificed after anesthesia (ketamine/chlorpromazine). Liver samples were maintained on ice for the lipid peroxidation assay and fixed in formalin and embedded in paraffin for histology. The remaining liver tissue was immediately frozen in liquid nitrogen and stored at −80 °C [40].

All animals received care in accordance with the Portuguese Law on Experimentation with Laboratory Animals, which is based on the principles of laboratory animal care as adopted by the *EC Directive 86/609/EEC for* animal experiments.

### 4.2. Determination of Metabolic and Oxidative Stress Parameters

After a 15-h fast, animals were anesthetized with ketamine/chlorpromazine. Blood was taken by heart puncture for the determination of lipids, uric acid, and hepatic parameters. Glucose determinations, which are tolerance tests to glucose and fasting plasma lipids (total and HDL cholesterol, and triglycerides), were performed as previously described [15]. Urinary 8-hydroxydeoxyguanosine (8-OHdG), and plasma malondialdehyde (MDA) were evaluated using competitive ELISA (OXIS health Products, Portland, OR, USA) and HPLC [15], respectively.

### 4.3. Lipid Content

Liver triglycerides and cholesterol content were determined as previously described [40] after methanol-chloroform extraction [41].

### 4.4. Assay of Antioxidant Enzymes in Liver

All antioxidant enzyme activities were determined after hepatic tissue was homogenized with PBS at a pH of 7.0, as previously described [42]. GPx activity was determined according to the method of Lawrence and Burk [43]. The enzyme activity was calculated using the value of ε340 = 6220/M per cm and the result was expressed in units of nmol NADPH/min per mg protein [42].

GRd activity was determined according to the method of Bellomo et al. [44]. The enzyme activity was calculated using the value of ε340 = 6220/M per cm and the result was expressed in units of nmol NADPH/min per mg protein [42].

### 4.5. Western Blot Analysis

Nuclear proteins of liver tissues were extracted as described by Tian et al. [45] with some modifications [46]. For total protein determinations, tissues were homogenized in a standard fashion. Samples containing 50 μg of protein were loaded on to a 12% sodium dodecyl sulfate-polyacrylamide gel electrophoresis (SDS-PAGE) gel and it was run and electro-blotted onto nitrocellulose membrane. Blots were blocked in 5% skimmed nonfat milk in PBS for 1 h, treated overnight with antibody against Nrf2 (Santa Cruz Biotechnology, CA, USA) or TNF-α (Abcam, UK), and then incubated with alkaline phosphatase secondary antibodies for 1 h. Immunoblots were developed with an ECF Western blotting detection system (Amersham Biosciences).

### 4.6. Nrf2-Binding Competition Assay

Nuclear extracts were used for the evaluation of Nrf2-binding activity to immobilized ARE using a TransAM Nrf2 kit (Active Motif). Briefly, nuclear extracts from liver lysates were incubated in a 96-well plate containing the immobilized consensus Nrf2-binding site. Wells were washed three times and bound to Nrf2, which was detected by the Nrf2 antibody and secondary antibody conjugated with horseradish peroxide. The signal was detected spectrophotometrically at 450 nm [47].

### 4.7. Evaluation of Inflammation in the Liver

TNF-α was measured in liver homogenates using an enzyme-linked immunosorbent assay (Quantikine Rat TNF-α, R&D Systems Europe Ltd, UK), according to manufacturer’s instructions. The hepatic TNF-α concentration was expressed per total protein.

### 4.8. Hematoxylin/Eosin and Oil-Red O Staining

Liver samples were collected, fixed in 10% formalin buffered solution, cut into 3-mm sections, and stained with hematoxylin/eosin using standard techniques [48]. To determine hepatic lipid deposition, frozen sections of liver were stained with oil-red O and it was washed and counterstained with hematoxylin in a standard manner.

### 4.9. Scintigraphic Analysis

The liver function was evaluated after intravenous bolus injection of ^99m^Tc-N-(3-bromo-2,4,6-trimethylphenylcarbamoilmethyl 1-iminodiacetic acid (Mebrofenin), which was gradually taken up by the hepatocytes and eventually excreted via the biliary pathway without any change to its chemical structure [16,19]. The hepatic extraction fraction (HEF) was calculated using deconvolution analysis of the liver first pass curve coming from scintigraphic data [17,18].

### 4.10. Protein

Protein content was determined using a Bio-Rad protein assay kit.

### 4.11. Statistical Analysis

All data were analyzed by using standard computer programs (GraphPad Prism PC Software version 3.0, ANOVA) and are expressed as mean ± SEM. Significant differences were evaluated using one-way ANOVA followed by the Bonferroni post-hoc test for individual comparisons. *p* < 0.05 was considered significant.

## Figures and Tables

**Table 1:** Body weight, liver weight, and ratio, glucose, and lipid profile in Wistar and Goto-Kakizaki rats and in GK rats fed with a high fat diet with or without α-lipoic acid and soybean oil.

	Wistar	GK	HFD	SO	LA
Body Weight (g)	559.7 ± 28.8	358.5 ± 8.7 ***	410.9 ± 5.1 *** ^§^	428.8 ± 12.0 *** ^§^	433.9 ± 10.9 *** ^§§^
Liver Weight (g)	6.75 ± 0.6	4.84 ± 0.2 *	11.05 ± 0.2 *** ^§§§^	8.32 ± 0.6 ^§§§ φφ^	6.37 ± 0.8 ^§ φφφ^
Liver Weight/Body Weight (%)	1.19 ± 0.07	1.36 ± 0.19	2.71 ± 0.07 *** ^§§§^	1.91 ± 0.11 * ^φφ^	1.63 ± 0.19 ^φφφ^
FBG (mmol/L)	4.07 ± 0.1	6.39 ± 0.4 ***	8.42 ± 0.4 *** ^§§^	7.19 ± 0.3 ***	6.78 ± 0.3 *** ^φ^
BG 2h After a Load (mmol/L)	6.5 ± 0.6	18.45 ± 2.7 ***	23.26 ± 1.6 ***	19.9 ± 0.8 ***	20.2 ± 0.5 ***
Cholesterol (mmol/L)	2.42 ± 0.21	4.0 ± 0.66	18.27 ± 3.06 *** ^§§§^	3.77 ± 0.56 ^φφφ^	2.39 ± 0.27 ^φφφ^
Non-HDL cholesterol (mmol/L)	0.71 ± 0.09	1.76 ± 0.51	16.01 ± 3.0 *** ^§§§^	2.23 ± 0.48 ^φφφ^	1.11 ± 0.16 ^φφφ^
Triglycerides (mmol/L)	1.15 ± 0.16	1.81 ± 0.78	6.23 ± 0.96 *** ^§§§^	2.22 ± 0.42 ^φφφ^	1.32 ± 0.16 ^φφφ^

Data are expressed as mean ± SEM (n = 7 animals per group). BG—blood glucose, FBG—fasting blood glucose, GK—Goto-Kakizaki, HFD—high fat diet, LA—α-lipoic acid, SO—soybean oil. * *p* < 0.05, *** *p* < 0.001 versus Wistar rats; § *p* < 0.05, §§ *p* < 0.01, §§§ *p* < 0.001 versus GK control group; φ *p* < 0.05, φφ *p*< 0.01, φφφ *p* < 0.001 versus GK HFD group.

**Table 2:** Serum levels of albumin, total bilirubin, alanine aminotransferase, aspartate aminotransferase, alkaline phosphatase, gamma-glutamyltranspeptidase, and hepatic extraction fraction of Wistar and Goto-Kakizaki rats and in GK rats fed with a high fat diet with or without α-lipoic acid and soybean oil.

	Wistar	GK	HFD	SO	LA
Albumin (g/dL)	2.7 ± 0.14	2.9 ± 0.1	2.6 ± 0.04	2.7 ± 0.06	2.4 ± 0.04
T-Bilirubin (mg/dL)	0.18 ± 0.05	0.16 ± 0.0	0.14 ± 0.02	0.14 ± 0.04	0.18 ± 0.02
AST (U/L)	147.5 ± 2.9	178 ± 15.1 **	176 ± 7.4 *	154 ± 12.4	151 ± 8.7 ^φ^
ALT (U/L)	49.3 ± 3.3	43.6 ± 1.1	40.1 ± 4.3	27 ± 2.0 ***	37.5 ± 1.3 ***
AST/ALT	3.1 ± 0.2	3.9 ± 0.1	4.2 ± 0.2	5.7 ± 0.4 *** ^§§ φφ^	3.9 ± 0.2 ^##^
ALP (U/L)	73.8 ± 22.1	137.1 ± 10.5 *	157.3 ± 9.8 **	114.8 ± 7.8 ^φ^	125.2 ± 7.4 ^φ^
γGT (U/L)	1.5± 0.15	2.1±0.1 *	1.9±0.1 *	1.6±0.2	1.3±0.2 ^§§ φ^
HEF (%)	100.0 ± 1.2	100 ± 1.5	87 ± 2.9 * ^§^	94.9 ± 1.8 ^φ^	97.8 ± 1.74 ^φ^

Data are expressed as mean ± SEM (n = 7 animals per group). ALP—alkaline phosphatase, ALT—alanine aminotransferase, AST—aspartate aminotransferase, GK—Goto-Kakizaki, γGT—γ-glutamyltranspeptidase, HEF—hepatic extraction fraction, HFD-high fat diet, LA—α-lipoic acid, SO—soybean oil. * *p* < 0.05, ** *p* < 0.01, *** *p* < 0.001 versus Wistar rats; § *p* < 0.05, §§ *p* < 0.01 versus GK control group, φ *p* < 0.05, φφ *p* < 0.01 versus GK HFD group. ## *p* < 0.01 versus GK SO group.

**Table 3:** Effect of α-lipoic acid on hepatic antioxidant enzymes, systemic markers of oxidative stress, and uric acid levels on Goto-Kakizaki rats fed a high fat-diet compared with age-matched Wistar rats and diabetic GK rats.

	Wistar	GK	HFD	SO	LA
GPx (nmol/min/mg protein)	181.2 ± 3.5	156.4 ± 2.5 ***	143.2 ± 2.2 *** ^§§^	151.2 ± 4.5 *** ^φ^	183.3 ± 2.5 ^§§§ φφφ ###^
GRd (nmol/min/mg protein)	42.3 ± 2.1	35.2 ± 1.9 *	28.2 ± 1.2 *** ^§^	32.1 ± 2.5 *	39.5 ± 2.1 ^φφ^
MDA (μmol/L)	1.12 ± 0.1	1.5 ± 0.1 **	1.8 ± 0.1 ***	1.42 ± 0.2 *	1.11 ± 0.1 ^φφ^
8-OHdG (ng/24h)	149.28 ± 17.0	306.57 ± 23.76 ***	415.8 ± 74.19 *** ^§^	302.37 ± 42.34 **	5.63 ± 0.36 ^φφφ ###^
Uric acid (mg/dL)	1.2 ±0.1	1.6 ± 0.2 *	1.8 ± 0.2 **	1.8 ± 0.2 **	1.2 ± 0.1 ^φ #^

Data are expressed as mean ± SEM (n = 7 animals per group). 8-OHdG—Urinary 8-hydroxydeoxyguanosine, GK—Goto-Kakizaki, GPx—glutathione peroxidase, GRd—glutathione reductase, HFD—high fat diet, LA—α-lipoic acid, MDA—malondialdehyde, and SO—soybean oil. * *p* < 0.05, ** *p* < 0.01, *** *p* < 0.001 versus Wistar rats, § *p* < 0.05, §§ *p* < 0.01, §§§ *p* < 0.001 versus GK control group, φ *p* < 0.05, φφ *p* < 0.01, φφφ *p* < 0.001 versus GK HFD group. # *p* < 0.05, ### *p* < 0.001 versus GK SO group.

## Supplementary Materials

Supplementary materials can be found at http://www.mdpi.com/1422-0067/19/9/2706/s1.
