# Supplementary material for: Lipoic Acid Prevents High-Fat Diet-Induced Hepatic Steatosis in Goto Kakizaki Rats by Reducing Oxidative Stress Through Nrf2 Activation
Source: Int J Mol Sci. 2018 Sep 11;19(9):2706. doi: 10.3390/ijms19092706 (PMC6164246; doi:10.3390/ijms19092706)
Supplement: Supplementary file 1 [file ijms-19-02706-s001.pdf]

Abbreviations

8-OHdG - Urinary 8-hydroxydeoxyguanosine

ALP- Alkaline phosphatase

ALT- Alanine aminotransferase

AST- Aspartate aminotransferase

GK - Goto-Kakizaki

γGT- γ-Glutamyltranspeptidase

GPx- Glutathione peroxidase

GRd- Glutathione reductase

GSH –Glutathione

HEF- Hepatic extraction fraction

HFD - High fat diet

α-LA - α-Lipoic acid

MDA - Malondialdehyde

NADPH – Nicotinamide adenine dinucleotide phosphate

NASH- Non-alcoholic steatohepatitis

Nrf2 - Nuclear factor E2 (erythroid-derived 2)-related factor-2

SO – Soybean oil

TNF-α - Tumor necrosis factor-α
